# Fentanyl decreases arterial blood oxygen saturation more than furanylfentanyl in mice due to increased apnea

**DOI:** 10.1007/s00213-026-07092-x

**Published:** 2026-07-02

**Authors:** Catherine Demery, Sierra C. Moore, Kelsey E. Kochan, Mengchu Li, Thomas D. Prince, Benjamin M. Clements, Zhongqi Chen, Jessica R. Whitaker-Fornek, Erica S. Levitt, Jessica P. Anand, John R. Traynor

**Affiliations:** 1 Edward F. Domino Research Center, Department of Pharmacology, University of Michigan Medical School, 1150 W. Medical Center Dr, Ann Arbor, MI 48109, USA; 2 Department of Pharmaceutical Sciences, University of Michigan College of Pharmacy, 428 Church St, Ann Arbor, MI 48109, USA; 3 Department of Anesthesiology, University of Michigan Medical School, 1500 E. Medical Center Dr, Ann Arbor, MI 48109, USA; 4 Department of Medicinal Chemistry, University of Michigan College of Pharmacy, 428 Church St, Ann Arbor, MI 48109, USA

**Keywords:** Furanylfentanyl, Fentanyl, Opioid, Mu-opioid receptor, Respiratory depression, Plethysmography, Oxygen saturation, Overdose, Apneas, Mouse

## Abstract

**Rationale:**

Fatal opioid overdoses involving synthetic opioids have increased >10-fold over the past 8 years. Fentanyl, a potent synthetic opioid acting at the mu-opioid receptor (MOR), has largely replaced heroin as the predominant illicit opioid available. Various analogs have been synthesized from the fentanyl scaffold, including 2-furanylfentanyl, one of the most trafficked analogs from 2019 to 2024. Despite its prevalence, the in vivo effects of furanylfentanyl are not well characterized, especially regarding respiratory depression.

**Objectives:**

To characterize furanylfentanyl relative to fentanyl in vitro and compare the effects of both drugs in mouse models of antinociception and respiratory depression.

**Methods:**

The affinity and potency of fentanyl and furanylfentanyl were determined in CHO cells overexpressing MOR. Male and female CD-1 mice were administered (*i.p.*) fentanyl or furanylfentanyl prior to measuring antinociception (warm-water tail withdrawal), respiration (whole-body plethysmography), or oxygen saturation (pulse oximetry).

**Results:**

Furanylfentanyl behaves as a partial agonist in vitro relative to fentanyl and morphine but with similar affinity and potency at MOR. In mice, fentanyl and furanylfentanyl produced similar effects on breathing parameters (including rate and inspiration time) that were greater than those induced by morphine, yet only fentanyl caused a drastic, long-lasting depletion of blood oxygen saturation. The discrepancy between effects on breathing and oxygen saturation may be explained by a greater number of apneas induced by fentanyl.

**Conclusions:**

These results highlight the complexity of fentanyl-induced respiratory depression and indicate that recreational use of 2-furanylfentanyl in humans may pose a greater risk of overdose than morphine but lesser than fentanyl.

## Graphical Abstract

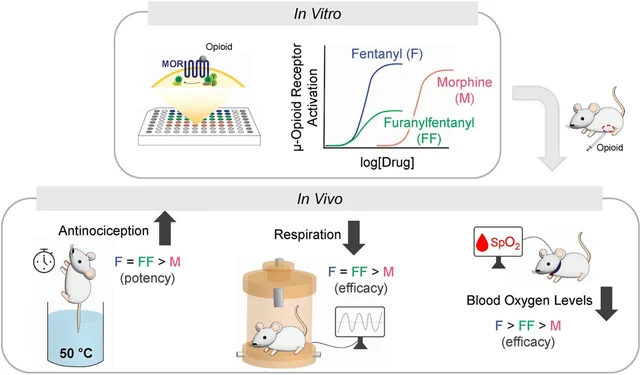

## Introduction

Over 100,000 people in the United States died of a drug overdose in 2023 (Ahmad et al. 2025). Deaths associated with synthetic opioids acting at the mu-opioid receptor (MOR), namely fentanyl and its analogs, have increased more than 10-fold over the past decade (Ahmad et al. 2025). Opioids are used clinically to relieve pain, an effect mediated through MOR; yet, activation of MOR also reduces breathing rate and regularity, causing CO_2_ retention and insufficient blood O_2_ levels that can trigger cardiorespiratory arrest (Bateman et al. 2023). Fentanyl induces respiratory depression more potently than morphine or heroin (Hill et al. 2020; Marchette et al. 2023). This is hypothesized to result from its greater potency at MOR (Traynor and Nahorski 1995) and greater induction of respiratory muscle rigidity (Cavallo et al. 2023). Furthermore, pharmacokinetic attributes of fentanyl enable rapid permeation of the blood-brain barrier (Kiyatkin 2019; Hill et al. 2020) yielding a rapid onset of action that both hinders the body’s chance to respond to rising CO_2_ levels and decreases the window of opportunity for reversal by administration of naloxone (Narcan^®^).

In addition to being more potent than heroin, fentanyl is easier to synthesize on a large scale (Cicero et al. 2015; Anderson 2017; Mars et al. 2019). As a result, fentanyl has largely replaced heroin as the most common illicit opioid available (Tupper et al. 2018; U.S. Drug Enforcement Administration 2025). Besides fentanyl, various fentanyl analogs have been identified in illicit drug seizures and overdose victims. 2-furanylfentanyl (furanylfentanyl, Fig. 1) is consistently detected in the U.S. illicit drug market (Commission 2021, 2022, 2023, 2024, 2025; Tennyson et al. 2021) and has been implicated in fatal overdoses worldwide (Helander et al. 2016; Guerrieri et al. 2017; Daniulaityte et al. 2019; O’Donnell et al. 2020). Despite the prevalence of furanylfentanyl, its effects are not well characterized (Nektaria Misailidi et al. 2017). Furanylfentanyl has a similar binding affinity to MOR as fentanyl but only shows partial agonism in vitro (Eshleman et al. 2020; Bilel et al. 2022; Anand et al. 2024). Yet, recent research has reported comparable antinociception (Bilel et al. 2022) and abuse liability (Chen et al. 2025; Obeng et al. 2025) between furanylfentanyl and fentanyl in rodent models. Whether furanylfentanyl induces respiratory depression akin to fentanyl remains unclear.

Herein, we compared the in vitro binding affinity and efficacy of furanylfentanyl, fentanyl, and morphine at MOR, as well as antinociception and respiratory depression. We confirmed that furanylfentanyl behaves as a partial agonist relative to fentanyl and morphine but with similar affinity and potency as fentanyl. In mice, furanylfentanyl significantly reduced blood oxygen levels as determined by pulse oximetry, although transiently and to a lesser extent than a 10-fold lower dose of fentanyl. Using whole-body plethysmography, furanylfentanyl produced similar changes in breathing parameters (rate, inspiration time and peak inspiratory flow) as fentanyl with approximately three-fold lower potency. Thus, doses of furanylfentanyl and fentanyl that induced comparable effects on breathing parameters produced markedly different effects on oxygen saturation. This discrepancy may be explained by a higher frequency of apneas induced by fentanyl (Demery et al. 2025) compared to its furanyl analog. This suggests another mechanism by which fentanyl produces more lethal effects in humans compared to other opioids.

## Methods

### Cell lines

Chinese hamster ovary (CHO) cells stably expressing the human MOR (MOR) were kindly provided by Dr. Lawrence Toll (Spagnolo et al. 2008) and used for competitive binding and [^35^S]GTPγS binding assays. Cells were incubated at 37 °C with 5% CO_2_ in 1:1 Gibco Dulbecco’s Modified Eagle’s Medium: F12 (Thermo Fisher Scientific). All media contained 10% fetal bovine serum (Millipore Sigma) and 5% penicillin/streptomycin (Thermo Fisher Scientific). Cells were grown to confluence before harvesting. PathHunter^®^ CHO cells expressing human MOR (DiscoveRx, 93–0213C2) were used to measure inhibition of adenyl cyclase and β arrestin-2 recruitment.

### Preparation of membrane fractions

Cells were incubated in warm harvesting buffer (150 mM NaCl, 20 mM HEPES, 0.50 mM EDTA, pH 7.4) for 15 min, then detached using a cell scraper and centrifuged for 3 min at 200 × *g*. The cell pellet was resuspended in ice-cold 50 mM Tris-HCl buffer (pH 7.4) and homogenized using a BioSpec Products Tissue-Tearor^™^ (Fisher Scientific) for 20 s on setting 4. The homogenate was then centrifuged for 20 min at 20,000 × *g* and 4 °C. The previous two steps were repeated except using the Tearor for 10 s on setting 2. The final pellet was resuspended in ice-cold 50 mM Tris-HCl, further homogenized in a glass douncer, then aliquoted and stored at −80 °C. A Pierce^™^ BCA Protein Assay Kit (Thermo Fisher Scientific) including bovine serum albumin as the standard was used to determine protein concentration.

### Competition binding

Competitive displacement of [^3^H]diprenorphine (Revvity; 250 μCi, 9.25 MBq) a non-selective opioid ligand, was used to measure drug affinity at MOR. Assays were carried out in 96-well plates with 10–20 μg membrane fraction protein, 0.20 nM [^3^H]diprenorphine, 50 mM Tris-HCl buffer (pH 7.4), and various concentrations of test ligand per well. Ligand concentration range used: 10 μM to 1.0 pM in 10-fold dilution steps. The plate was incubated for 1 h (to allow binding to reach equilibrium) at room temperature (RT) on a shaker. Next, samples were filtered through Whatman^®^ Grade CF/C glass filters (Millipore Sigma) and washed several times with 50 mM Tris-HCl buffer (pH 7.4). Filters were saturated with EcoLume^™^ Liquid Scintillation Cocktail (MP Biomedical) and then radioactivity was quantified using a 2450 MicroBeta^2®^ Microplate Counter (Revvity). Buffer was used to determine total binding and 10 μM naloxone (Fisher Scientific) was used to determine non-specific binding. Results presented are the average ± S.E.M. of three independent assays performed on different days; within each assay, duplicates were performed and averaged. Data were fit to a one-site competition binding curve (non-linear regression) using GraphPad Prism 10.0.0. The Cheng-Prusoff equation was used to convert the resulting IC_50_ values to K_i_ values (Cheng and Prusoff 1973).

### [^35^S]GTPγS binding

Agonist ability to stimulate [^35^S]guanosine-5’-O-(γ-thio) triphosphate ([^35^S]GTPγS; Revvity; 1250 Ci/mmol) binding at MOR was determined. Assays were carried out in 96-well plates with 10–20 μg membrane fraction protein, 0.10 nM [^35^S]GTPγS, assay buffer (50 mM Tris-HCl, 100 mM NaCl, 10 mM MgCl_2_, 1 mM EDTA, pH 7.4), 30 μM guanosine 5’-diphosphate (Millipore Sigma), and various concentrations of test ligand per well. Ligand concentration range used: 10 μM to 1.0 pM in 10-fold dilution steps. The plate was incubated for 1 h at RT on a shaker. Next, samples were filtered through Whatman^®^ Grade CF/C glass filters (Millipore Sigma) and washed several times with assay buffer. Radioactivity was quantified as described earlier. Assay buffer was used to determine baseline stimulation and test agonists were compared to 10 μM (D-Ala^2^, *N*-Me-Phe^4^, Gly^5^-ol)-enkephalin (DAMGO; Millipore Sigma). Results presented are the result of 3–5 independent assays performed on different days; within each assay, duplicates were performed and averaged. Data were fit to a sigmoidal dose-response curve for agonist stimulation (non-linear regression) using GraphPad Prism 10.0.0.

### β Arrestin-2 recruitment

β arrestin-2 recruitment to human MOR expressed in PathHunter^®^ CHO cells (DiscoverX 93–0213C2) was determined using the Beta-Glo^®^ β-galactosidase Enzyme-Complementation Assay (Promega). Cells were prepared per manufacturer instructions and then incubated (37 °C with 5% CO_2_) with various concentrations of orthosteric agonist for 60 min. Agonist concentration range used: 10 μM to 1.0 pM in 10-fold dilution steps. Cells were allowed to cool to RT (15 min) then administered chemiluminescent Beta-Glo^®^ Detection Reagent and incubated for 60 min in the dark at RT. β-galactosidase enzyme activity, indicating β arrestin-2 recruitment, was detected as luminescence measured via a GloMax^®^ Explorer Microplate Reader (Promega). Agonist activity was compared to 10 μM DAMGO.

### Inhibition of adenylyl cyclase

Varying concentrations of orthosteric agonist were used to inhibit cAMP production by adenyl cyclase as described by the cAMP-Glo^™^ Assay Protocol (Promega). All incubations were done at RT. Briefly, PathHunter^®^ CHO cells stably expressing MOR (DiscoverX 93–0213C2) were plated in 96-well plates and incubated in induction buffer for 15 min. Various concentrations of agonist (10 μM to 1.0 pM in 10-fold dilution steps) were added and incubated for 15 min. To evaluate agonist-induced inhibition of adenyl cyclase, 10 μM forskolin (activator of adenylyl cyclase) was added and incubated for 15 min. Lysis buffer was then added and the plate was incubated for 30 min on a shaker. Next, cAMP Detection Solution was added and the plate was incubated for 20 min, followed by addition of Kinase-Glo^®^ Reagent and a final 10-min incubation in the dark. Activity was detected by luminescence measured with a GloMax^®^ Explorer Microplate Reader (Promega). Maximum possible effect was considered 10 μM DAMGO with minimum effect as forskolin only (no agonist).

### Drugs

The following compounds were obtained from Cayman Chemical Company: fentanyl hydrochloride (item # 14719), 2-furanylfentanyl hydrochloride (item # 18705), and naloxone hydrochloride (item # 15594). Morphine sulfate powder was obtained from Spectrum Chemical Mfg. Corp. (item # M1167) and naloxone methiodide powder was purchased from Millipore Sigma (item # N129). All compounds were dissolved in saline. Each animal received one intraperitoneal (*i.p.*) injection at 10 mL/kg of body weight.

### Animal husbandry and care

Adult male and female CD-1 mice (5–12 weeks of age) were used for all experiments unless otherwise specified. Mice were bred in-house at the University of Michigan or purchased from Envigo (Indianapolis, IN). Mice were maintained on a 12-hour light/dark cycle, with experiments occurring during the light phase. Mice were housed in clear polypropylene cages with corncob bedding, in groups of 2–5 by sex, with access to food, water, and enrichment *ad libitum*. All experiments were conducted in accordance with the Guide for the Care and Use of Laboratory Animals (Committee 2011) and approved by the University of Michigan Institutional Animal Care and Use Committee.

### Warm-water tail withdrawal

The antinociceptive effect of various agonists was determined using the warm water tail withdrawal (WWTW) assay. Experimenter was blinded to the drug treatments given. CD-1 mice were restrained in a plastic cylinder with tails submerged 2–3 cm into a 50 °C water bath. Latency to tail flick was recorded with a 20 s cutoff to prevent tissue damage. Dose-response curves were created using a cumulative dosing procedure. Briefly, mice received an *i.p.* injection of saline, and baseline latency was recorded 15 min later. Next, mice received an *i.p.* injection of 0.1 mg/kg furanylfentanyl or fentanyl, and latency time was re-measured after 15 min. The previous step was repeated for increasing doses of furanylfentanyl or fentanyl up to a 1.0 mg/kg cumulative dose. For morphine, which has a slower onset and longer duration of action, 30-min dosing intervals were employed. Fentanyl and furanylfentanyl groups, *n* = 12 mice each; morphine and saline groups, *n* = 6 mice each. Excel and GraphPad Prism 10.0.0 were used for all analyses.

### MouseOx

The MouseOx^®^ Plus Small Animal Pulse Oximeter (Starr Life Sciences, Oakmont, PA) was used to measure pulse oximetry parameters in awake, freely moving CD-1 mice. (The fur of CD-1 mice is white, so they do not need to be shaved for accurate infrared sensing.) Mice were habituated to enclosures with a practice collar for 1 h. The practice collar was replaced with the MouseOx^®^ collar and then baseline pulse oximetry measurements, including percent oxygen saturation (SpO_2_), were recorded for 1 h using MouseOx^®^ software version 1.6.8. At t = 0, mice were injected (*i.p.*) with opioid. Data was recorded for 100 min post-injection and averaged into 5 min bins.

### Whole-body plethysmography

Respiratory parameters of CD-1 mice were recorded in whole-body plethysmography (WBP) chambers (SCIREQ vivoFlow^®^, Montreal, QC Canada) supplied with 0.5 L/min of medical air USP (Cryogenic Gases). Mice were acclimated to WBP chambers for 30 min on day one. On day two, mouse weights and temperatures (Braun digital infrared thermometer) were recorded, and body temperature (35–37 °C), chamber temperature (20–22 °C) and chamber humidity (10%) were used in volume calculations. Mice were placed in chambers for 30 min prior to injection: 10 min of habituation (data not saved) and 20 min of baseline recording. Mice were removed for intraperitoneal injection of opioid or saline and then returned to chambers for 60 min post-injection. For naloxone reversal studies, mice were removed 15 min post-injection of opioid for *i.p.* naloxone administration, then returned to chambers for 45 min post-naloxone. Data were recorded with Emka Technologies iox 2.10.8.6 software and exported in 30-sec bins to determine the following plethysmography parameters: breathing rate (breaths per min), inspiration (Ti) time, expiration time (Te), peak inspiratory flow (PIF), peak expiratory flow (PEF), end expiratory pause (EEP), tidal volume (TV), and expired volume (EV). For apnea analysis, session recordings were replayed in Emka Technologies iox 2.10.8.6 software to generate and export the data with single-breath bins. Further data processing for all parameters is described in the Data Analysis section.

### Hot plate

Latency to nociceptive behaviors (i.e., forepaw licking or jumping) in mice on a 55 °C hot plate was measured at baseline, 15 min after *i.p.* injection of 3.2 mg/kg furanylfentanyl, and subsequently every 5 min after *i.p.* injection of a rescue agent (3.2 mg/kg naloxone, 45 mg/kg naloxone methiodide, or saline as control). Experimenter was blinded to the drug rescue given. A cut-off time of 1 min was implemented to prevent tissue damage.

### Data analysis

For all comparisons, *p* < 0.05 was considered significant. Post-hoc analyses were only considered for significant ANOVAs or mixed effects analyses and were performed to evaluate all possible multiple comparisons. Measures of affinity (K_i_), potency (EC_50_), maximum effect, and area under the curve were compared using a one-way ANOVA. Antinociceptive t_1/2_ values were compared via unpaired t-test. For all plethysmography parameters (ex., breathing rate, inspiration time, tidal volume, etc.), data were averaged into 5-min bins for analysis, normalized to each mouse’s average baseline value (t=−15–0 min), and groups were compared using a two-way ANOVA or mixed effects model (with treatment and time as independent variables). If significant, then a one-way ANOVA with Tukey’s post-hoc test was used to compare group averages from 10 to 15 min post-injection; this same method was applied for blood oxygen saturation. For apnea analysis, data were exported as individual breaths. Excel was used to calculate the number of apneas per group from 0 to 15 min post-injection using the following criteria: Apnea=[Te+EEP]>2[Avg.Te+EEP,post-injection], with the average length of Te + EEP from t = 0–60 min used to define an apnea for each mouse individually. The number of apneas and apnea frequency $\left(Freq.\;=\frac{\#\;\mathrm{apneas}}{\#\;\mathrm{breaths}}\right)$ per treatment group from t = 0–15 min post-injection were compared via one-way ANOVA with Tukey’s post-hoc test. GraphPad Prism 10.0.0 was used for all statistical analyses.

## Results

### Furanylfentanyl is a potent partial agonist at the mu-opioid receptor

We first examined the properties of fentanyl and furanylfentanyl at MOR in vitro compared to morphine (Table 1). Fentanyl, furanylfentanyl, and morphine all bound with comparable single-digit nanomolar affinity to MOR (*p* = 0.102). Fentanyl (*p* = 0.013) and furanylfentanyl (*p* = 0.011) were significantly more potent than morphine in stimulating [^35^S] GTPγS binding; furanylfentanyl shower lower efficacy (20% maximum activation of the standard MOR full agonist, DAMGO) compared to fentanyl and morphine (*p* < 0.0001 for both). In evaluating agonist ability to inhibit adenylyl cyclase, we found that both fentanyl (*p* < 0.0001) and furanylfentanyl (*p* < 0.0001) were significantly more potent than morphine. All three agonists reached the same maximum effect in this assay. This is unsurprising, as adenylyl cyclase is downstream of G protein signaling and therefore subject to signal amplification. In other words, lower efficacy agonists (i.e., furanylfentanyl) can still achieve complete inhibition of adenylyl cyclase, so the ceiling effect makes it difficult to distinguish between full and partial agonists in this assay.

Agonist recruitment of β arrestin-2, a multifunctional protein that typically drives receptor internalization but can act as a scaffold for other signaling molecules (Seyedabadi et al. 2021), was also determined. We observed that fentanyl (*p* = 0.003) and furanylfentanyl (*p* = 0.002) were also more potent than morphine in this assay. Maximum β arrestin-2 recruitment (relative to DAMGO) differed by agonist: fentanyl (65%; *p* < 0.0001 vs. furanylfentanyl, morphine) > morphine (35%) > furanylfentanyl (17%; *p* = 0.0006 vs. morphine).

### Furanylfentanyl induces comparable thermal antinociception to fentanyl

We next evaluated the ability of furanylfentanyl to induce thermal antinociception in mice, as measured by the warm-water tail withdrawal assay. A cumulative dosing procedure was employed (Fig. 1A), with 15-min dosing intervals for fentanyl and furanylfentanyl, and 30-min dosing intervals for morphine due to its slower onset (Hill et al. 2020). As shown in Fig. 1B, fentanyl (*p* < 0.0001) and furanylfentanyl (*p* < 0.0001) were significantly more potent in affording antinociception than morphine. There was no difference in antinociceptive potency (EC_50_) between fentanyl and furanylfentanyl (*p* = 0.628). However, the effect of fentanyl persisted for a significantly longer time (Fig. 1C; *p* < 0.0001).

### Fentanyl decreases blood oxygen saturation in mice to a greater extent than furanylfentanyl

We next considered whether furanylfentanyl would decrease oxygen saturation in vivo. Blood oxygen saturation in the carotid artery was monitored in mice using pulse oximetry (Fig. 2A). At all doses tested (10, 32, and 100 mg/kg, *i.p.*), morphine did not reduce oxygen saturation compared to saline (Fig. 2B). In contrast, doses of 3.0 and 10 mg/kg (*i.p.*) fentanyl markedly reduced oxygen saturation for an extended period (Fig. 2C). Furanylfentanyl transiently decreased blood oxygen saturation (Fig. 2D). Comparing the peak effect, doses of 10 (*p* = 0.0010) and 32 mg/kg *i.p.* furanylfentanyl (*p* < 0.0001) significantly reduced blood oxygen saturation relative to saline (Fig. 2E). Fentanyl depleted blood oxygen saturation more potently and to a greater extent than furanylfentanyl: doses of 3.0 and 10 mg/kg *i.p.* produced significantly lower blood oxygen saturation than 32 mg/kg *i.p.* furanylfentanyl (*p* = 0.0005 vs. 3.0, *p* < 0.0001 vs. 10 mg/kg fentanyl). The effect of 10 mg/kg fentanyl on blood oxygen saturation was fully reversed by the antagonist naloxone, confirming an opioid receptor-mediated action (Fig. 2F).

### Fentanyl and furanylfentanyl produce similar changes in breathing parameters in mice

We next asked whether the more severe and prolonged decrease in oxygen saturation caused by fentanyl compared to furanylfentanyl could be explained by differences in breathing parameters, as determined using whole-body plethysmography in awake, freely moving animals (Fig. 3A). The effects of fentanyl (0.32, 1.0, 3.2, and 10 mg/kg *i.p.*) and furanylfentanyl (0.32, 1.0, 3.2, 10, and 32 mg/kg *i.p.*) administration were examined. As morphine did not reduce oxygen saturation at any dose tested, only the highest dose (100 mg/kg *i.p.*) was included in the plethysmography experiments. The average breathing rate by group at t = 15 min post-injection (compared via 1-way ANOVA with post-hoc analyses) showed that doses of 3.2 (*p* = 0.015) and 10 (*p* = 0.024) mg/kg fentanyl, as well as 32 mg/kg furanylfentanyl *i.p.* (*p* = 0.0008), decreased breathing rate relative to saline (Fig. 3B, bar graph).

As depicted in Fig. 3A, a given breath is comprised of inspiration time (Ti) and expiration time (Te); thus, if breathing rate slows, either Ti and/or Te should lengthen. Ti is the most sensitive marker of opioid-induced respiratory depression (Demery et al. 2025), and we observed a clear dose-dependent increase in Ti for both fentanyl and furanylfentanyl (Fig. 3C). Doses of 3.2 (*p* = 0.001) and 10 (*p* < 0.0001) mg/kg fentanyl, along with 10 (*p* = 0.007), and 32 (*p* < 0.0001) mg/kg furanylfentanyl *i.p.*, significantly increased Ti relative to saline at t = 15 min post-injection (Fig. 3C, bar graph). The volume of air inhaled (tidal volume) depends on Ti and peak inspiratory flow (PIF), which describes the fastest flow of air through the lungs in each breath. Doses of 1 (*p* = 0.01) and 10 (*p* = 0.021) mg/kg fentanyl, as well as 10 (*p* = 0.004), and 32 (*p* < 0.0001) mg/kg furanylfentanyl *i.p.* decreased peak inspiratory flow relative to saline at t = 15 min post-injection (Fig. 3D, bar graph). Only 10 mg/kg fentanyl increased tidal volume compared to control (*p* = 0.001), while both 3.2 (*p* = 0.009) and 10 mg/kg fentanyl (*p* < 0.0001) increased tidal volume relative to 32 mg/kg furanylfentanyl (Supplementary Fig. 2B). In addition, the effects of 100 mg/kg *i.p.* morphine on various breathing parameters, generally similar to those produced by 1.0 mg/kg *i.p.* fentanyl, were not different from saline at t = 15 min post-injection.

Opioid effects on expiration were minimal (Supplementary Fig. 1). Expiration time and end expiratory pause (EEP; a delay between the end of one breath and the beginning of the next) were transiently increased by 10 mg/kg fentanyl at 5 min post-injection (*p* < 0.0001 for both metrics), and 2) EEP was increased by 32 mg/kg *i.p.* furanylfentanyl from t = 5–15 min post-injection (*p* ≤ 0.001 for all timepoints; Supplementary Fig. 1B, 1C). Last, 3.2 (*p* = 0.007) and 10 mg/kg fentanyl (*p* = 0.008) increased peak expiratory flow relative to 32 mg/kg furanylfentanyl *i.p.* (Supplementary Fig. 1D).

### Fentanyl induces apneas that correlate with blood oxygen loss

Changes in breathing parameters did not explain the more drastic and prolonged decrease in saturated oxygen induced by 3.2 and 10 mg/kg fentanyl compared to 32 mg/kg furanylfentanyl (Fig. 2C, D and E). Thus, we questioned whether there might be a difference in the number of apneas caused by the two drugs, as apneas have been shown to decrease oxygen saturation in humans (Yadollahi et al. 2010; Wali et al. 2020) and we have shown apneas correlate with reductions in blood oxygen saturation (Demery et al. 2025). A severe apnea following 10 mg/kg fentanyl is displayed in Fig. 4B. Shown in Fig. 4C are representative breathing traces from saline-, 10 mg/kg fentanyl-, 32 mg/kg furanylfentanyl-, and 100 mg/kg *i.p.* morphine-treated mice. As shown in Fig. 4D, significantly more apneas were observed in mice administered 10 mg/kg fentanyl (*p* = 0.004), with a similar trend in the 3.2 mg/kg fentanyl group (*p* = 0.078), compared to saline during the first 15 min following opioid administration. Apnea frequency, % apneas out of total breaths, was also significantly increased by 10 mg/kg fentanyl (*p* = 0.003) during the first 15 min post-injection (Fig. 4E). Duration of apneas by treatment group is shown in Supplementary Fig. 3, along with representative Te + EEP distributions (t = 0–15 min). To investigate an association between oxygen depletion (Fig. 4F) and frequency of apneas, the Pearson correlation between AUC for blood oxygen saturation loss (t = 0–15 min) and the number of apneas (t = 0–15 min) was determined (*p* = 0.007; Fig. 4G). Likewise, there was a significant correlation between blood oxygen saturation loss and apnea frequency (*p* = 0.004; Fig. 4H). We also chose to look at the components of apneas separately and found that Te but not EEP correlated with oxygen saturation depletion (Supplementary Fig. 3); however, the correlation was less strong than for apneas. These results indicate that there is a correlation between frequency of apneas and blood oxygen depletion following administration of opioids, as we have shown previously (Demery et al. 2025).

### Effects of fentanyl and furanylfentanyl on breathing were readily reversed by naloxone

The current standard of care for opioid overdose is naloxone, or Narcan^®^. Therefore, we evaluated the ability of two doses of naloxone (1.0 and 3.2 mg/kg *i.p.*, chosen based on previous work (Anand et al. 2024), to reverse the effects of fentanyl and furanylfentanyl (3.2 mg/kg *i.p.* each) on breathing rate, inspiration time, and peak inspiratory flow. Rescue injections were administered 15 min after opioid (Fig. 5A). Either dose of naloxone readily reversed the effects of 3.2 mg/kg fentanyl and furanylfentanyl on breathing rate, inspiration time, and peak inspiratory flow within 5 min of rescue injection (Fig. 5B, C and D), with the exception that 1.0 mg/kg naloxone did not rescue the furanylfentanyl-induced decrease in PIF.

To determine if any of the actions on breathing have a peripheral component, as recently reported (Ruyle et al. 2025), we examined furanylfentanyl reversal using 45 mg/kg *i.p.* naloxone methiodide, considered to be a peripherally restricted antagonist. Based on previously published literature showing that doses of 30–100 mg/kg *i.p.* naloxone methiodide could reverse the respiratory effects of opioid agonists (Lewanowitsch et al. 2006), as well as our in vitro data showing that naloxone methiodide has a 15-fold lower affinity for MOR than naloxone (K_i_ values of 15.24 and 0.72 nM, respectively), the dose of 45 mg/kg was chosen. Naloxone methiodide did not significantly rescue the effects of either opioid on any breathing parameter within the first 5 min post-rescue injection (Fig. 5B, C, D). However, by 10 min post-administration, naloxone methiodide reversed the effects of furanylfentanyl on inspiration time and peak inspiratory flow (bar graphs not shown). The slower onset of effect may reflect the different pharmacokinetic properties of naloxone methiodide relative to naloxone. As naloxone methiodide is permanently charged, it will have more difficulty crossing the blood brain barrier than naloxone, and so any centrally mediated effects would be delayed. To examine whether the effects of this dose of naloxone methiodide were truly peripherally restricted, we performed the hot plate test and found that naloxone methiodide at 45 mg/kg *i.p.* reversed furanylfentanyl-induced antinociception (Supplementary Fig. 4). Since nociceptive responses in this test involve higher brain centers, this suggests that, at the dose used, naloxone methiodide likely permeated the central nervous system.

## Discussion

Herein, we have shown that furanylfentanyl and fentanyl produce similar effects on breathing rate, inspiration time, and peak inspiratory flow in mice, with furanylfentanyl exhibiting approximately three-fold lower potency than fentanyl. On the other hand, fentanyl markedly decreases blood oxygen saturation relative to the small, transient effect of furanylfentanyl. The greater reduction in blood oxygenation following fentanyl administration may result from the higher frequency of apneas produced by fentanyl.

Both fentanyl and furanylfentanyl were more potent than morphine in G-protein activation and β arrestin-2 recruitment at the mu-opioid receptor. Furanylfentanyl behaved as a lower efficacy agonist relative to fentanyl in both assays, consistent with other literature (Eshleman et al. 2020; Bilel et al. 2022; Anand et al. 2024). Yet, the low partial agonist activity of furanylfentanyl in vitro was sufficient to produce substantial effects in vivo. In mice, furanylfentanyl induced thermal antinociception with comparable potency to fentanyl, although the antinociceptive duration of activity was shorter. Fentanyl and furanylfentanyl have previously been shown to produce equipotent mechanical antinociception (Bilel et al. 2022), and comparable abuse liability (Chen et al. 2025; Obeng et al. 2025), which explains the recreational use of furanylfentanyl in humans. On the other hand, furanylfentanyl administration induces less severe precipitated withdrawal symptoms than fentanyl in mice (Gannon et al. 2025; Obeng et al. 2025), suggesting nuanced characteristics of this potent partial agonist in vivo.

At higher doses than those required to achieve antinociception, furanylfentanyl transiently decreased blood oxygen saturation, though the effect was of much lower magnitude than fentanyl, even at a 10x greater dose. Both 3.2 and 10 mg/kg *i.p.* fentanyl drastically reduced blood oxygen saturation to approximately 50% within 15 min. Of note, this experiment measured arterial oxygen saturation (SpO_2_); blood oxygen levels also depend on the hemoglobin content of the blood and partial pressure of O_2_ (PaO_2_). Considering the oxygen dissociation curve (SpO_2_ vs. PaO_2_) is non-linear, larger decreases in PaO_2_ are needed to lower SpO_2_, so it’s possible that other drug treatments may have caused below-threshold PaO_2_ depletion. It is worth mentioning that pulse oximetry relies on a pulse, so hypotension or motion artifacts from skeletal muscle activity could impact the results. The limited effect of furanylfentanyl on SpO_2_ agrees with a previous study reporting that furanylfentanyl did not reduce oxygen saturation at doses up to 6 mg/kg *i.p.* (Bilel et al. 2022), measured as average drug effect over a 2-hour period. Given the reports of human fatalities involving furanylfentanyl (Helander et al. 2016; Guerrieri et al. 2017; Daniulaityte et al. 2019; O’Donnell et al. 2020), the transient decrease in blood oxygen saturation we observed in mice may translate to a more severe effect in humans, as mice can better adapt to changes in blood oxygen due to their higher specific metabolic rate (Demetrius 2005; Perlman 2016; Arias-Reyes et al. 2021).

Surprisingly, the difference in blood oxygen saturation following fentanyl and furanylfentanyl administration was not explained by differences in the suppression of breathing rate or inspiration. As the two fentanyls produced similar effects on breathing metrics derived from plethysmography, albeit fentanyl 3–10x more potently, these observations do not explain the greater effect on blood oxygen levels induced by fentanyl. Rather, we found that the number of apneas was associated with blood O_2_ levels, consistent with our previous work (Demery et al. 2025) and in line with findings that apneas reduce oxygen saturation in humans (Findley et al. 1983; Yadollahi et al. 2010; Wali et al. 2020). Apneas are aberrantly long cessations in breathing relative to the breathing pattern of the individual at the time. Apneas both increase CO_2_ and decrease O_2_; normally, CO_2_ accumulation triggers an increase in ventilatory drive, but opioids suppress this protective chemoreflex (Dahan et al. 2001). Thus, longer apneas may prevent sufficient gas exchange and reduce arterial oxygen saturation, especially given they occur in the context of opioid-induced reductions in breathing rate and flow. Apnea, beyond hypoventilation, can promote atelectasis (i.e., alveolar collapse), and intrapulmonary shunting of blood, potentially worsening hypoxia (Fletcher et al. 1990).

It is also possible that other opioid-induced effects may have contributed to the hypoxia observed. Opioids decrease body temperature and oxygen consumption (Lotti et al. 1966; Rosow et al. 1980), and increase respiratory muscle rigidity, constricting air flow (Cavallo et al. 2023; Neumueller et al. 2023). Opioids also decrease blood pressure (McClellan et al. 2023), which could affect the alveolar-arterial (A-a) gradient. Multiple studies have shown that opioids increase the alveolar-arterial (A-a) gradient, indicating ventilation-perfusion mismatch and impaired O_2_ exchange in the lung (Getsy et al. 2025), although this may occur on a slower time course than decreases in oxygen saturation (Neumueller et al. 2023). Indeed, opioids reduce fluid clearance in the lungs (Guney et al. 2025), as do conditions of hypoxia (Taenaka and Matthay 2025). Thus, multiple respiratory perturbations may contribute to the loss in oxygen saturation observed following fentanyl or furanylfentanyl administration.

Morphine was even less effective at reducing blood oxygen than furanylfentanyl despite its higher in vitro efficacy. This is likely a reflection of morphine’s slower onset, which allows central chemoreceptors to compensate for rising CO_2_ levels in the blood to better stabilize blood gases (Bateman et al. 2023) – a reflex that would be hindered by the more rapidly acting fentanyl and furanylfentanyl. Together, these results highlight the complexity of the mechanisms underlying fentanyl’s lethality, which is not clearly delineated based on plethysmography parameters alone.

We found that naloxone at 3.2 mg/kg *i.p.* was readily able to rescue changes in breathing parameters caused by 3.2 mg/kg *i.p.* fentanyl or furanylfentanyl. On the other hand, a previous study in mice under hypercapnic conditions found that 3.0 mg/kg *i.p.* naloxone was necessary to rescue the effects of 0.15 mg/kg *i.p.* fentanyl on breathing (Hill et al. 2020), suggesting a much higher naloxone-to-fentanyl dosage requirement. However, the effects of opioids are exacerbated under conditions of hypercapnia, potentially explaining the discrepancy. In light of recent research demonstrating peripheral opioid receptor contribution to opioid-induced respiratory depression (Ruyle et al. 2025), we investigated the rescue potential of naloxone methiodide, a peripherally restricted opioid receptor antagonist. We found that it was able to attenuate respiratory depression induced by furanylfentanyl but with a slower onset of effect than naloxone. However, naloxone methiodide, at the same dose and on the same time course, also blocked antinociception in the hot plate assay, typically regarded as a supraspinal response. This suggests that the observed reversal of furanylfentanyl-induced changes in breathing was due to naloxone methiodide acting centrally. Indeed, naloxone methiodide has been detected in the brains of rats after *i.v.* administration (Kiyatkin et al. 2026). Nonetheless, other studies reporting that naloxone methiodide reversed opioid-induced respiratory depression and antinociception in mice have suggested this is peripherally mediated (Lewanowitsch et al. 2006; He et al. 2009), so we cannot rule out a peripheral component to the rescue effects of naloxone methiodide shown here.

In addition, opioid effects vary by strain and species (Robson et al. 1985; Boorman et al. 2025). The present study was performed in mice, while the study by Ruyle et al. 2025 utilized rats; thus, species differences could contribute to the inconsistence in findings. However, both central and peripheral effects of naloxone methiodide have been shown in rats and mice. Admittedly, there are limitations of using a mouse model to probe the risk of respiratory depression and overdose in humans. Namely, mice are more resistant to changes in blood oxygen saturation because of their higher specific metabolic rate (Demetrius 2005; Perlman 2016; Arias-Reyes et al. 2021), and mice can tolerate much higher mg/kg doses of drugs, in part due to 20x faster turnover of their blood volume than humans (Shin et al. 2010). On the other hand, breathing control networks and sites of modulation by opioids, as well as opioid signaling, are well-conserved in mice (Ramirez et al. 2021), as is the rank-order of opioid potency (Knotkova et al. 2009; Minami et al. 2009).

In conclusion, we have shown that furanylfentanyl reduces oxygen saturation much less than fentanyl at doses that produce comparable reductions in breathing rate and inspiration. Since both drugs have similar effects on breathing, albeit that fentanyl is 3x more potent, the differential effects could be explained by the greater number of apneas induced by fentanyl in the first 15 min post-injection, during which time oxygen levels in furanylfentanyl- but not fentanyl-treated mice recover. It is possible that inducing apneas requires higher opioid efficacy than depressing breathing rate or inspiration, reflecting the lower efficacy of furanylfentanyl relative to fentanyl observed in vitro. These findings underscore the complexities of fentanyl-induced respiratory depression while also contributing to our understanding of opioid-induced respiratory phenotypes. Overall, our results indicate that the recreational use of 2-furanylfentanyl in humans poses a significant overdose risk that is greater than that of morphine but lesser than that of fentanyl.

## Supplementary Material

Supplementary

**Supplementary Information** The online version contains supplementary material available at https://doi.org/10.1007/s00213-026-07092-x.

## Figures and Tables

**Fig. 1:**
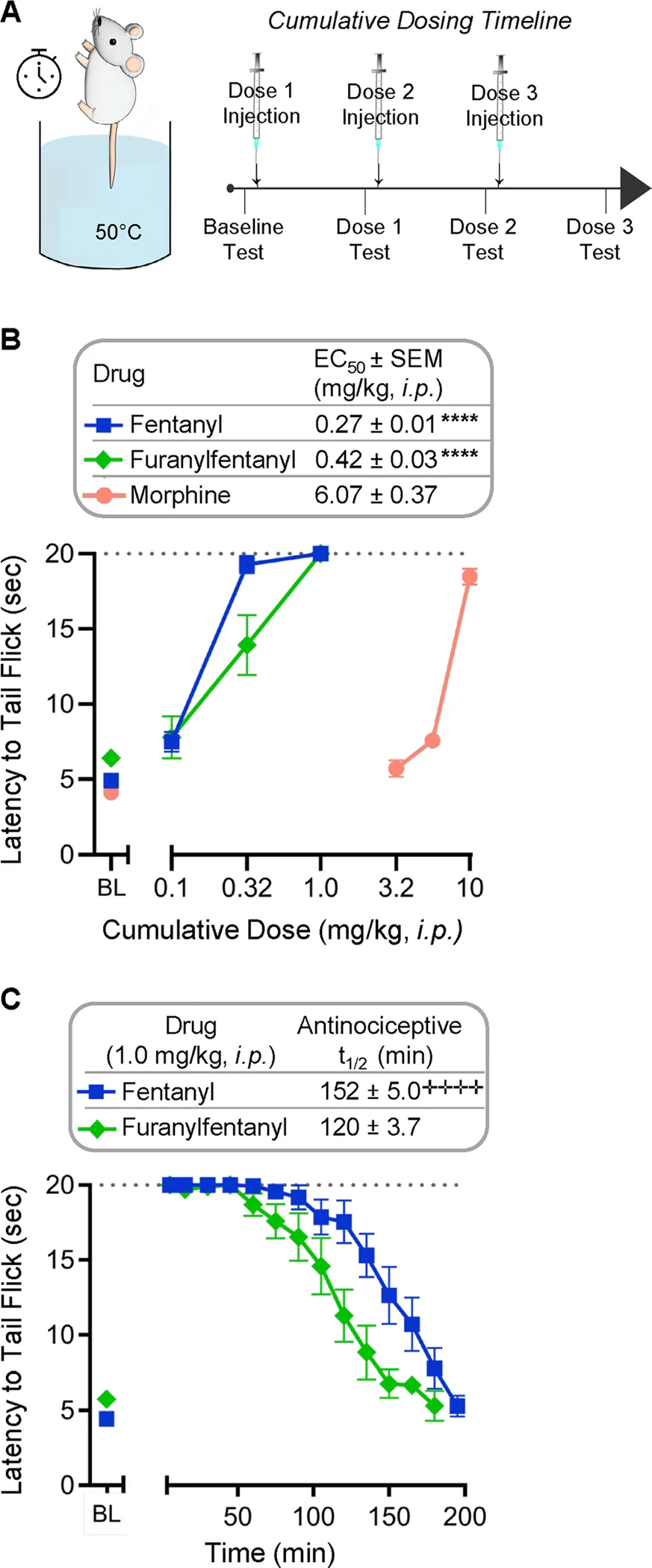
Furanylfentanyl induces comparable thermal antinociception to fentanyl. (**A**) Male and female CD-1 mice were restrained with their tails dipped into 50 °C water. Latency to tail flick (avg. ± SEM) was recorded with a maximum allowed time of 20 s. Cumulative dosing was performed as described in the Methods section, with 15-min dosing intervals for fentanyl and furanylfentanyl, and 30-min dosing intervals for morphine and saline. (**B**) Dose-response curves for fentanyl (*n*=12), furanylfentanyl (*n*=12), and morphine (*n*=6). EC_50_ values were compared via 1-way ANOVA (F(2,27)=508.3, *p*<0.0001) with Tukey’s post-hoc test (p-values displayed). Repeated saline injections did not alter latency from baseline and are not shown. (**C**) Duration of analgesic action for 1.0 mg/kg *i.p.* fentanyl (*n*=12) and furanylfentanyl (*n*=12). Antinociceptive t_1/2_ was defined as the time at which antinociceptive activity had dropped to half of the max. effect; values were compared via unpaired t-test (t=5.188, df=21, *p*<0.0001). ****p<0.0001 vs. morphine; ^⊹⊹⊹⊹^*p*<0.0001 vs. furanylfentanyl

**Fig. 2:**
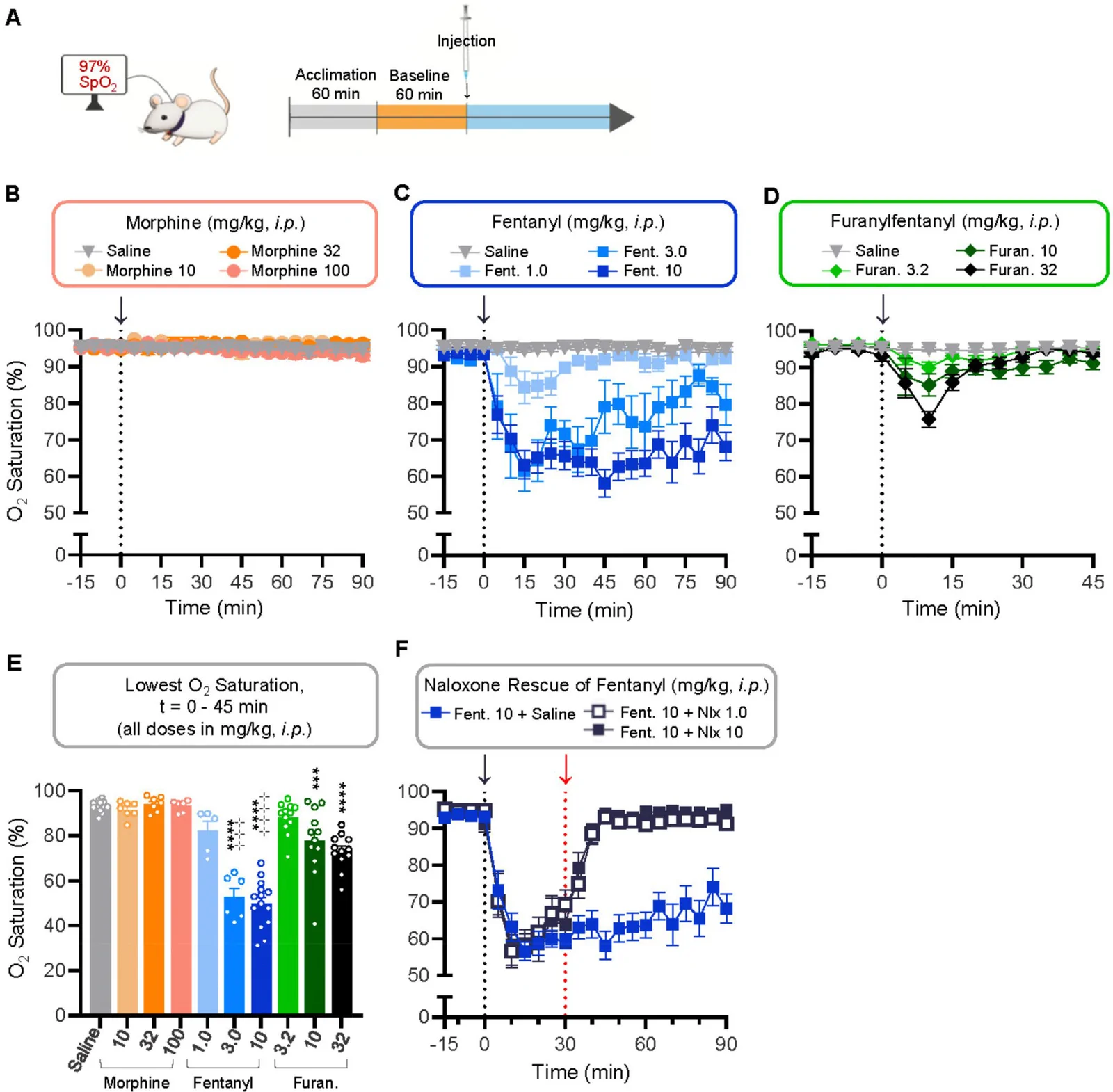
Furanylfentanyl decreases oxygen saturation to a lesser extent than fentanyl. (**A**) Blood oxygen saturation of male and female CD-1 mice following *i.p.* injection of opioid or saline (black arrows) was monitored using the PulseOx^®^ system. (**B**-**D**) Data are presented as average ± SEM. All doses of all drugs were compared via mixed-effects analysis from t=0–45 min (main effect of treatment, F(9,85)=41.97, *p*<0.0001; main effect of time, F(3.156,257.7)=10.41, *p*=0.0289; time x treatment interaction: F(81,735)=4.259, *p*<0.0001). (**B**) Effect of morphine on oxygen saturation at 10 (*n*=7), 32 (*n*=6), and 100 (*n*=6) mg/kg from 0–90 min post-injection. (**C**) Effect of fentanyl on oxygen saturation at 1.0 (*n*=5), 3.0 (*n*=6), and 10 (*n*=14) mg/kg from 0–90 min post-injection. (**D**) Effect of furanylfentanyl on oxygen saturation at 3.2 (*n*=12), 10 (*n*=12), and 32 (*n*=12) mg/kg from 0–45 min post-injection. (**E**) Lowest O_2_ saturation by drug treatment (average ± SEM) from t=0–45 min post-injection was compared via 1-way ANOVA (F(9,85)=35.70, *p*<0.0001) with Tukey’s post-hoc test (p-values displayed). (**F**) Oxygen saturation (average ± SEM) following reversal of 10 mg/kg fentanyl with saline (*n*=14), 1.0 (*n*=12) or 10 (*n*=11) mg/kg naloxone administered 30 min after fentanyl (i.p., red arrow). All 10 treatment groups were compared from t=30–90 min via mixed-effects analysis (main effect of treatment, F(2,36)=65.85, *p*<0.0001; main effect of time, F(5.505,187.7)=2.500, *p*=0.0276, time x treatment interaction: F(22,375)=1.853, *p*=0.0117). ***p<0.001, ****p<0.0001 relative to saline; ^⊹⊹⊹⊹^*p*<0.0001 relative to 32 mg/kg furanylfentanyl (only shown for fentanyl)

**Fig. 3:**
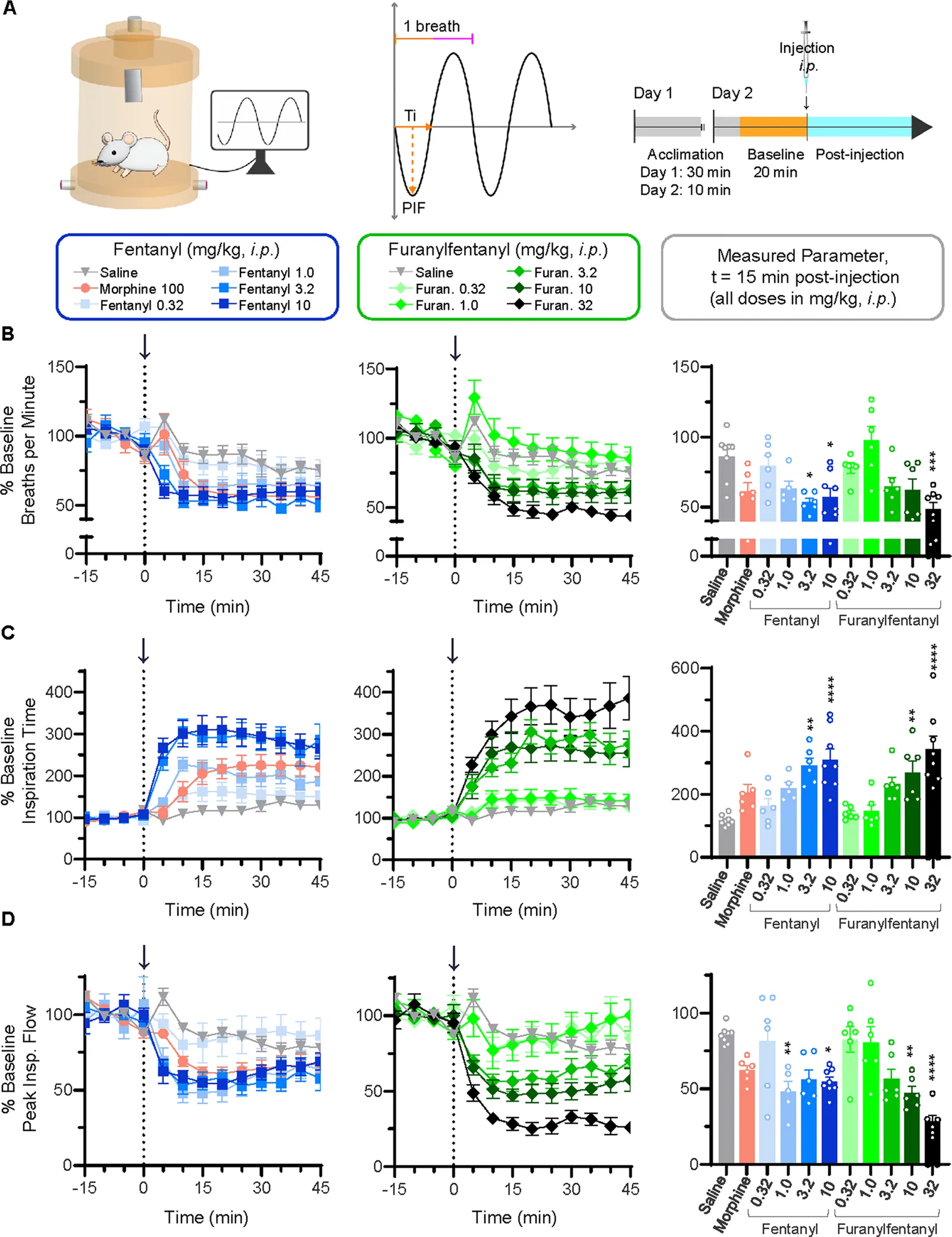
Furanylfentanyl and fentanyl produce similar changes in breathing parameters. (**A**) Male and female CD-1 mice (*n*=8 for saline, 10 mg/kg fentanyl, and 32 mg/kg furanylfentanyl; *n*=6 for all other groups) were placed in whole-body plethysmography chambers (vivoFlow^®^) supplied with air. Various respiratory parameters were measured using EMKA Technologies software. (**B-D**) Arrows and dotted lines in all time course graphs represent *i.p.* injection point. Data were normalized to each mouse’s baseline and are shown as treatment group average ± SEM. (**B**) Breaths per minute by treatment group from t=0–45 min were compared via mixed-effects analysis (main effect of treatment, F(10,61)=5.802, *p*<0.0001; main effect of time, F(5.727,345.8)=73.15, *p*<0.0001; time x treatment interaction, F(80,483)=2.164, *p*<0.0001). Average values by treatment group from t=10–15 min post-injection were compared via 1-way ANOVA (F(10,60)=5.875, *p*<0.0001) with Tukey’s post-hoc test (p-values shown on bar graph). (**C**) Inspiration time by treatment group from t=0–45 min was compared via mixed-effects analysis (main effect of treatment, F(10,61)=10.39, *p*<0.0001; main effect of time, F(3.766,227.9)=34.67, *p*<0.0001; time x treatment interaction, F(80,484)=2.604, *p*<0.0001). Average values by treatment at t=10–15 min were compared via 1-way ANOVA (F(10,60)=8.208, *p*<0.0001) with Tukey’s post-hoc test (p-values shown in bar graph). (**D**) Peak inspiratory flow by treatment group from t=0–45 min was compared via mixed-effects analysis (main effect of treatment, F(10,59)=8.571, *p*<0.0001; main effect of time, F(4.605,268.8)=23.54, *p*<0.0001; time x treatment interaction, F(80,467)=2.033, *p*<0.0001). Average values by treatment at t=10–15 min were compared via 1-way ANOVA (F(10,58)=7.600, *p*<0.0001) with Tukey’s post-hoc test (p-values shown in bar graph). *p<0.05, **p<0.01, ***p<0.001, ****p<0.0001 vs. saline

**Fig. 4:**
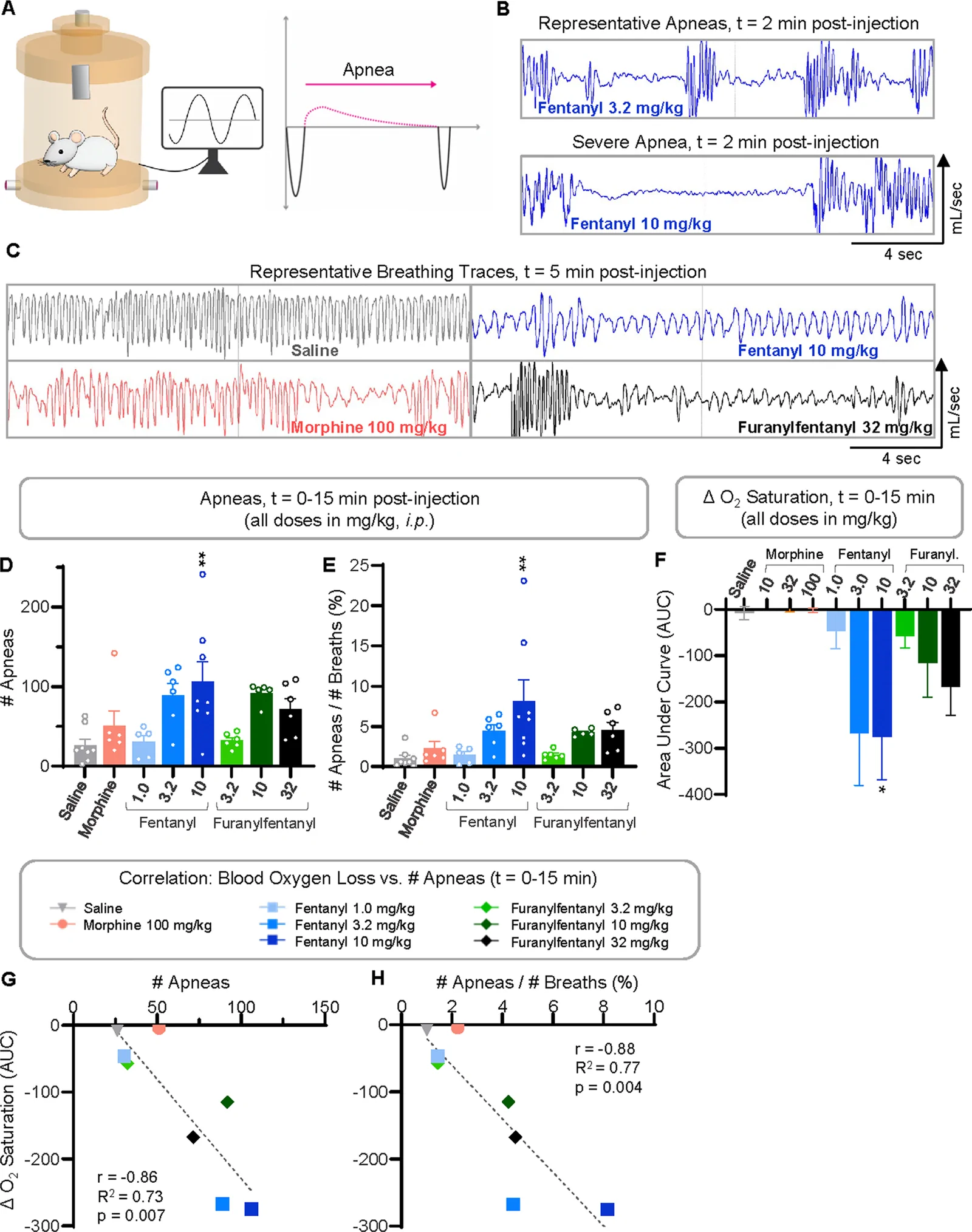
Fentanyl induces apneas that correlate with blood oxygen loss. (**A**) Plethysmography data was examined breath-by-breath for apnea analysis in mice injected *i.p.* with saline (*n*=8), 100 mg/kg morphine (*n*=6), furanylfentanyl (3.2 mg/kg, *n*=6; 10 mg/kg, *n*=5; 32 mg/kg, *n*=6), or fentanyl (1.0 mg/kg, *n*=5; 3.2 mg/kg, *n*=6; 10 mg/kg, *n*=8). An apnea was defined as a breath with (Te+EEP) > 2*[Te+EEP]_avg_, with [Te+EEP]_avg_ equal to each mouse’s average Te+EEP in the post-injection period. (**B**) Traces showing representative apneas (top) and a severe apnea (bottom) 2 min after 3.2 mg/kg or 10 mg/kg fentanyl injection, respectively. (**C**) Representative 20-sec breathing traces are shown for selected treatments at approximately t=5 min post-injection. (**D**) Number of total apneas (average ± SEM) from t=0–15 min post-injection by treatment was compared using 1-way ANOVA (F(7,42)=4.436, *p*=0.0009) with Tukey’s post-hoc test (p-values displayed). (**E**) Apneic frequency, defined as the % apneas out of total breaths, by treatment from t=0–15 min (average ± SEM) was compared via 1-way ANOVA (F(7,42)=3.743, *p*=0.0031) with Tukey’s post-hoc test (p-values displayed). (**F**) Saturated oxygen loss (AUC) from t=0–15 min by group (average ± SEM) using data from Fig. 2 was compared via 1-way ANOVA (F(9,85)=2.784, *p*=0.0066) with Tukey’s post-hoc test (p-values displayed). (**G**) Pearson correlation between # total apneas (Fig. 4D) and oxygen saturation loss (Fig. 4F) from t=0–15 min post-injection. (**H**) Pearson correlation between apneic frequency (Fig. 4E) and blood oxygen loss (Fig. 4F) from t=0–15 min post-injection. *p<0.05, **p<0.01 vs. saline

**Fig. 5:**
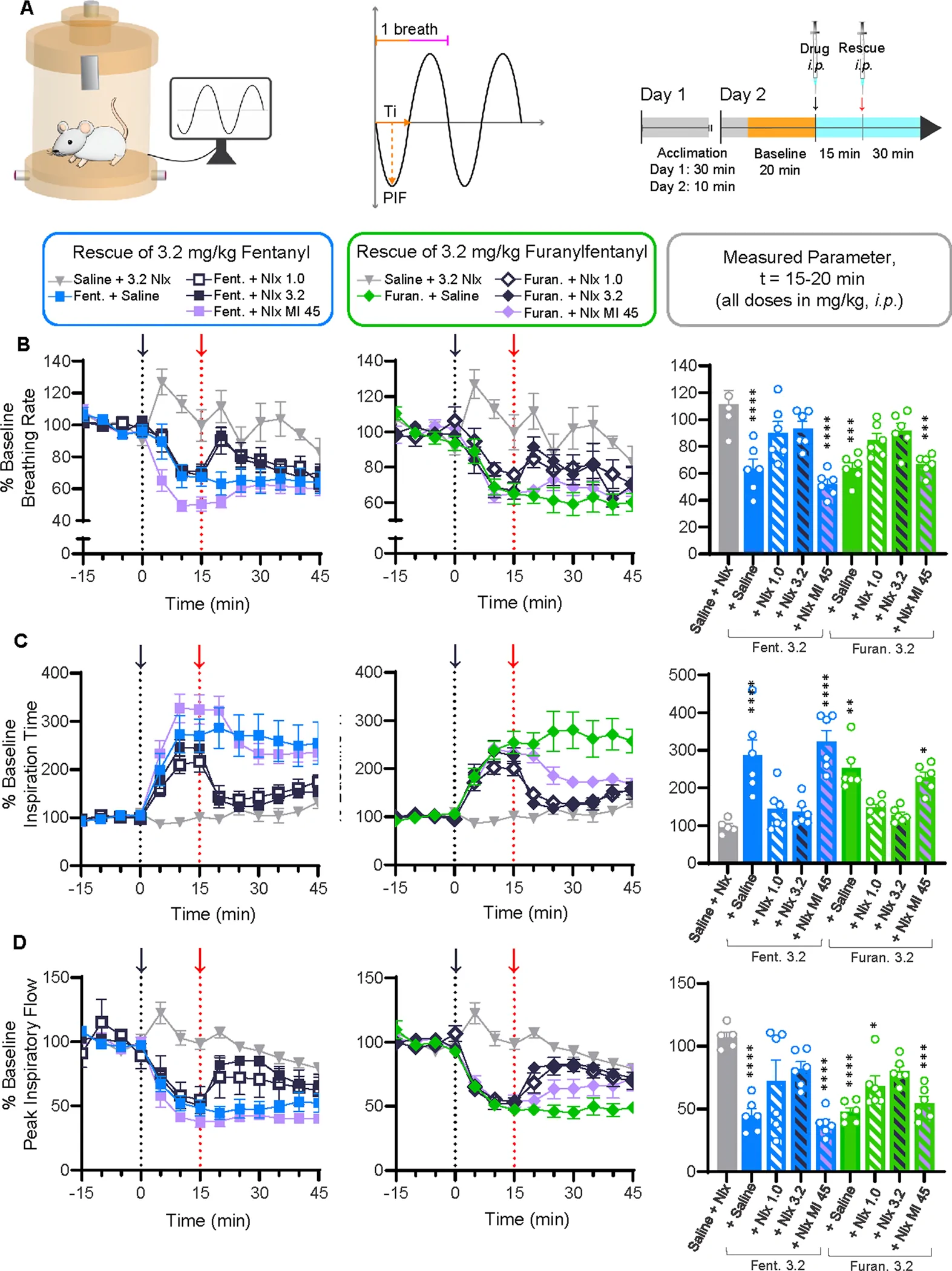
Effects of fentanyl and furanylfentanyl on breathing were readily reversed by naloxone. (A) The ability of naloxone (Nlx; 1.0 or 3.2 mg/kg *i.p.*) or naloxone methiodide (Nlx MI; 45 mg/kg *i.p.*) to reverse the effects of opioid administration on breathing parameters was evaluated in male and female CD1 mice (*n*=6 per group) using plethysmography according to the timeline shown. (**B-D**) Arrows and dotted lines in all time course graphs represent *i.p.* injection of opioid or saline at t=0 min (grey) and rescue agent or saline at t=15 min (red). Data were normalized to each mouse’s baseline and are shown as treatment group average ± SEM. (**B**) Breathing rate of all 9 treatment groups was compared via mixed-effects analysis from t=15–45 min (main effect of treatment, F(8,45)=4.048, *p*=0.0011; main effect of time, F(4.128,183.3)=12.30, *p*<0.0001; time x treatment interaction, F(40,222)=2.440, *p*<0.0001). Breathing rate by treatment was compared from 0–5 min post-rescue injection (t=15–20 min; bar graph) via 1-way ANOVA (F(8,44)=9.554, *p*<0.0001) with Tukey’s post-hoc test (p-values displayed). (**C**) Inspiration time (Ti) of all 9 treatment groups was compared via mixed-effects analysis from t=15–45 min (main effect of treatment, F(8,45)=8.718, *p*<0.0001; main effect of time, F(2.855,126.8)=6.348, *p*=0.0006; time x treatment interaction, F(40,222)=4.531, *p*<0.0001). Ti by treatment was compared from 0–5 min post-rescue injection (t=15–20 min; bar graph) via 1-way ANOVA (F(8,44)=12.43, *p*<0.0001) with Tukey’s post-hoc test (p-values displayed). (**D**) Peak inspiratory flow (PIF) of all 9 treatment groups was compared via mixed-effects analysis from t=15–45 min (main effect of treatment, F(8,45)=7.556, *p*<0.0001; main effect of time, F(2.133,94.71)=3.900, *p*=0.0213; time x treatment interaction, F(40,222)=2.210, *p*=0.0002). PIF by treatment was compared from 0–5 min post-rescue injection (t=15–20 min; bar graph) was compared via 1-way ANOVA (F(8,44)=8.386, *p*<0.0001) with Tukey’s post-hoc test (p-values displayed). *p<0.05, **p<0.01, ***p<0.001, ****p<0.0001 vs. saline + 3.2 mg/kg naloxone

**Table 1:** In vitro properties of fentanyl, furanylfentanyl, and morphine at the human mu-opioid receptor (MOR). Structures and cLogP were derived from ChemDraw. Using membrane fractions from CHO cells overexpressing MOR, binding affinity (K_i_) was measured by competitive displacement of 0.2 nM [^3^H]diprenorphine (*n*=3 per drug); K_i_ values were not significantly different per 1-way ANOVA (F(2,6)=3.590). G protein activation was determined by agonist-induced [^35^S]GTPγS binding and accumulation (*n*=3 for morphine and furanylfentanyl, *n*=5 for fentanyl). EC_50_ values and % maximum/max. effect by drug were independently compared via 1-way ANOVA (EC_50_: F(2,8)=9.646, *p*=0.0074; % max. effect: F(2,8)=106.4, *p*<0.0001) with Tukey’s post-hoc test (p-values shown in table). In live CHO cells overexpressing MOR, inhibition of adenylyl cyclase was determined using the cAMP-Glo^™^ Assay (*n*=3 per drug). EC_50_ values and % max. effect were compared independently via 1-way ANOVA (EC_50_: F(2,6)=437.3, *p*<0.0001; % max. effect: F(2,6)=4.385, non-significant) with Tukey’s post-hoc test (EC_50_ only; p-values shown in table). β arrestin-2 recruitment was measured via the Beta-Glo^®^β-galactosidase Enzyme-Complementation Assay (*n*=3 per drug). EC_50_ values and % max. effect were compared independently via 1-way ANOVA (EC_50_: F(2,6)=23.36, *p*=0.0015; % max. effect: F(2,6)=213.5, *p*<0.0001) with Tukey’s post-hoc test (p-values shown in table). For all assays, 10 μM DAMGO was considered maximum effect.

	Fentanyl	Furanyl-fentanyl	Morphine

Structure	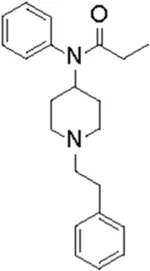	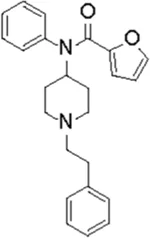	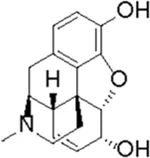

cLogP	3.6	3.5	0.6

**Binding Affinity**			
K_i_ ± SEM (nM)	1.9 ± 0.6	1.3 ± 0.1	4.2 ± 1.3

**G-protein Activation**			
EC_50_ ± SEM (nM)	* 27.2 ± 5.8	* 9.3 ± 2.2	146.7 ± 48.3
Max. Effect^a^ ± SEM (%)	⊹⊹⊹⊹ 90.8 ± 3.8	20.4 ± 3.4	⊹⊹⊹⊹ 98.5 ± 3.8

**Adenylyl Cyclase Inhibition**			
EC_50_ ± SEM (nM)	**** 3.9 ± 0.3	**** 6.6 ± 0.6	49.3 ± 2.0
Max. Effect^a^ ± SEM (%)	105.5 ± 2.5	98.5 ± 3.4	116.3 ± 6.1

**β arrestin-2 Recruitment**			
EC_50_ ± SEM (nM)	** 75.0 ± 12.8	** 53.2 ± 12.0	399.7 ± 67.3
Max. Effect^a^ ± SEM (%)	**** ⊹⊹⊹⊹ 65.2 ± 2.1	*** 17.1 ± 1.7	35.1 ± 1.0

* *p*<0.05,

** *p*<0.01,

*** *p*<0.001,

**** *p*<0.0001 vs. morphine;

⊹⊹⊹⊹ *p*<0.0001 vs. furanylfentanyl

## Data Availability

All data supporting the findings of this study are available within the paper and its Supplementary Information.
