# Supplementary material for: Fentanyl decreases arterial blood oxygen saturation more than furanylfentanyl in mice due to increased apnea
Source: Psychopharmacology (Berl). Author manuscript; Available in PMC 2026 Oct 5. (PMC13636089; doi:10.1007/s00213-026-07092-x)
Supplement: Supplementary file 1 [file NIHMS2209832-supplement-Supplementary.docx]

**PSYCHOPHARMACOLOGY JOURNAL**

**SUPPLEMENTARY MATERIAL FOR:**

**Fentanyl decreases arterial blood oxygen saturation more than furanylfentanyl in mice due to increased apnea**

Catherine Demery^1^, Sierra C. Moore^1^, Kelsey E. Kochan^1^, Mengchu Li^1^, Thomas D. Prince^1^, Benjamin M. Clements^1^, Zhongqi Chen^2^, Jessica R. Whitaker-Fornek^1^, Erica S. Levitt^1,3^, Jessica P. Anand^1^*, and John R. Traynor^1,4^*

^1^Edward F. Domino Research Center, Department of Pharmacology, University of Michigan Medical School, 1150 W. Medical Center Dr., Ann Arbor, MI, 48109, USA

^2^Department of Pharmaceutical Sciences, University of Michigan College of Pharmacy, 428 Church St., Ann Arbor, MI, 48109, USA

^3^Department of Anesthesiology, University of Michigan Medical School, 1500 E. Medical Center Dr., Ann Arbor, MI 48109, USA

^4^Department of Medicinal Chemistry, University of Michigan College of Pharmacy, 428 Church St., Ann Arbor, MI, 48109, USA

*Co-communicating authors

Communicating Authors:

John R. Traynor, PhD

[jtraynor@umich.edu](mailto:jtraynor@umich.edu)

Jessica P. Anand, PhD

[janand@umich.edu](mailto:janand@umich.edu)
